# Mechanisms of implementing public health interventions: a pooled causal mediation analysis of randomised trials

**DOI:** 10.1186/s13012-018-0734-9

**Published:** 2018-03-12

**Authors:** Hopin Lee, Alix Hall, Nicole Nathan, Kathryn L. Reilly, Kirsty Seward, Christopher M. Williams, Serene Yoong, Meghan Finch, John Wiggers, Luke Wolfenden

**Affiliations:** 1 Centre for Statistics in Medicine, Nuffield Department of Orthopaedics Rheumatology and Musculoskeletal Sciences, University of Oxford, Oxford, United Kingdom; 2 School of Medicine and Public Health, University of Newcastle, Newcastle, New South Wales Australia; 3 Neuroscience Research Australia, Sydney, New South Wales Australia; 4 Hunter Medical Research Institute, Newcastle, New South Wales Australia; 5 Hunter New England Population Health, Newcastle, New South Wales Australia

**Keywords:** Mechanism, Theoretical domains framework, Public health, Implementation science, Mediation analysis

## Abstract

**Background:**

The World Health Organization recommends that nations implement evidence-based nutritional guidelines and policies in settings such as schools and childcare services to improve public health nutrition. Understanding the causal mechanism by which implementation strategies exert their effects could enhance guideline implementation. The aim of this study was to assess the mechanisms by which implementation strategies improved schools and childcare services’ adherence to nutrition guidelines.

**Methods:**

We conducted a mechanism evaluation of an aggregated dataset generated from three randomised controlled trials conducted in schools and childcare services in New South Wales, Australia. Each trial examined the impact of implementation strategies that targeted Theoretical Domains Framework constructs including knowledge, skills, professional role and identity, environmental context and resources. We pooled aggregated organisation level data from each trial, including quantitative assessments of the Theoretical Domains Framework constructs, as well as measures of school or childcare nutrition guideline compliance, the primary implementation outcome. We used causal mediation analysis to estimate the average indirect and direct effects of the implementation strategies and assessed the robustness of our findings to varying levels of unmeasured and unknown confounding.

**Results:**

We included 121 schools or childcare services in the pooled analysis: 79 allocated to receive guideline and policy implementation strategies and 42 to usual practice. Overall, the interventions improved compliance (odds ratio = 6.64; 95% CI [2.58 to 19.09]); however, the intervention effect was not mediated by any of the four targeted Theoretical Domains Framework constructs (average causal mediation effects through knowledge = − 0.00 [− 0.05 to 0.04], skills = 0.01 [− 0.02 to 0.07], professional role and identity = 0.00 [− 0.03 to 0.03] and environmental context and resources = 0.00 [− 0.02 to 0.06]). The intervention had no significant effect on the four targeted Theoretical Domains Framework constructs, and the constructs were not associated with school or childcare nutrition guideline compliance. Potentially, this lack of effect could be explained by imprecise measurement of the mediators. Alternatively, it is likely that that the interventions were operating via alternative mechanisms that were not captured by the four Theoretical Domains Framework constructs we explored.

**Conclusions:**

Even though public health implementation strategies led to meaningful improvements in school or childcare nutrition guideline compliance, these effects were not mediated by key targeted constructs of the Theoretical Domains Framework. Future research should explore the mechanistic role of other Theoretical Domains Framework constructs and evaluate system-level mechanisms informed by an ecological framework.

**Trial registration:**

All trials were prospectively registered with the Australian New Zealand Clinical Trials Registry (ACTRN12613000543785. Registered 15/05/2013; ACTRN12614001148662. Registered 30/10/2014; ACTRN12615001032549. Registered 1/10/2015).

**Electronic supplementary material:**

The online version of this article (10.1186/s13012-018-0734-9) contains supplementary material, which is available to authorized users.

## Background

Nutrition risk factors are the leading causes of the global disease burden [1]. Consequently, United Nations member states declared 2016–2025 as the decade of action on nutrition [2]. Dietary guidelines provide evidence-informed recommendations regarding the dietary patterns recommended for optimal health and well-being and to reduce the risk of dietary-related chronic diseases [3]. The World Health Organization has recommended that nations implement evidence-based nutritional guidelines and policies in settings such as schools and childcare services to improve public health nutrition [4]. School and childcare-based nutritional guidelines typically make recommendations regarding the types of foods and beverages that should be provided (or made available to children) and in quantity, variety and frequency [5, 6]. A considerable challenge to achieving such objectives is the limited evidence base regarding the effectiveness of implementation strategies [7, 8]. Reviews of strategies that aim to improve the implementation of nutrition policies in schools and childcare services have identified few trials and report strategies that achieved equivocal effects [9, 10].

Understanding the causal mechanism by which implementation strategies exert their effects can improve the impact of strategies to enhance guideline implementation [7]. Approaches to improve policy or practices consistent with guideline recommendation are often multi-strategic and target a range of intermediary factors (or mediators) that are hypothesised to be causally be linked to successful implementation [7, 11]. The effectiveness of such strategies may be improved by retaining (or strengthening) strategies that target mediators which cause improvements in guideline implementation. Strategies could also be refined by discarding intervention components that target mediators that do not cause improvements in guideline implementation, or those that fail to shift important mediators [7, 12]. Despite the importance of understanding mechanisms of effect, few mechanistic evaluations of implementation strategies exist, and to our knowledge, none have been conducted on trials of strategies to improve implementation of nutrition guidelines.

Recent methodological advances have developed robust analytical techniques to quantify the extent to which of intervention effects are channelled through selected mediating variables. These new methods are based on clearly outlined counterfactual definitions of causal effects along with explicit assumptions required for making causal inferences [13–15]. However, conducting mechanistic evaluations of pragmatic trials in settings such as schools and childcare services is particularly challenging as guideline implementation typically occurs at the organisational level. Often, an insufficient sample of organisations is recruited to allow for sufficient power to undertake mediation analyses. There is also a lack of agreement as to which constructs should be tested as possible mediators of implementation and how they should be measured [16].

In this exploratory study, we aimed to overcome power limitations by aggregating data from three homogeneous implementation trials in schools and childcare services and used a theory-driven consensus approach to identify key constructs that could plausibly mediate the effects of implementation strategies on nutrition policy uptake. Thus, the overarching objective of this study was to quantify the extent to which selected Theoretical Domains Framework (TDF) constructs mediate the effect of implementation strategies on nutrition policy uptake in schools and childcare services.

## Methods

### Design and data sources

We conducted a mechanism evaluation by aggregating data from three homogeneous randomised controlled trials. The primary aims of all three trials were to increase compliance with state-wide nutrition guidelines regarding the nutritional quality of foods offered to children via a school canteen or provided as part of a food service at childcare. All trials were conducted in the Hunter New England Region of New South Wales, Australia, by the Hunter New England Population Health Research Group, a partnership between the University of Newcastle and a government population health unit [17]. Each trial randomly allocated schools or childcare services to receive a multicomponent complex implementation intervention or usual care. The outcomes of two trials (CAFÉ [18] and SNACS [19]) are published, and one trial (BMI [20]) has closed data collection. For each trial, independent reviewers assessed their quality using the Cochrane Risk of Bias Tool [21] (Additional file 1). Key characteristics of all three trials are described in Table 1. Detailed information about each trial are available in published reports and trial protocols [18–20].

**Table 1:** Characteristics of trials

	CAFÉ [15]	SNACS [16]	BMI [17]
Aim	To assess the impact of an audit and feedback intervention on improving rural and remote schools’ compliance with the NSW Healthy School Canteen Policy.	To assess the effectiveness of a theoretically designed multi-strategy intervention in increasing the implementation of a healthy canteen policy in Australian primary schools.	To assess the effectiveness of a multi-strategy intervention to improve the implementation of nutrition guidelines in childcare services.
Design	Randomised controlled trial	Randomised controlled trial	Randomised controlled trial
Intervention	Main intervention component was menu audit, and subsequent provision of feedback via a written report and telephone call up to 4 times over a 12-month period.	Multi-component intervention conducted over a 9-month period, including the following; executive support, canteen manager/parent training, (5 h group training workshop) tools and resources, on-going support, performance monitoring and feedback (written canteen menu report), and recognition.	Intervention 1: provision of training and resources, menu planning checklists, recipe ideas and budgeting fact sheets, audit and feedback. Intervention 2: provision of training and resources, menu planning checklists, recipe ideas and budgeting fact sheets, audit and feedback, ongoing support via support officer providing expert advice and assistance to facilitate guideline implementation. Each intervention service received two face-to-face contacts, following the menu planning workshop. The implementation support officer, the service manager and cook signed a memorandum of understanding which outlined each party’s responsibilities working to improve food service.
Control	Usual care––access to NSW Government programs directed at supporting school promotion of healthy eating and physical activity.	Usual care––access to NSW Government programs directed at supporting school promotion of healthy eating and physical activity.	Usual care and hard copy of the Caring for Children resource.
Primary outcome	(i) The proportion of schools with a canteen menu that did not include red or banned foods and beverages and (ii) the proportion of schools where green items make up the majority of the menu defined as more than 50% of listed menu items.	(i) The proportion of schools with a canteen menu that did not include red or banned foods and beverages and (ii) the proportion of schools where green items make up the majority of the menu defined as more than 50% of listed menu items.	Compliance with guideline recommendations, defined as one that provides 50% of the recommended daily serves of each of the Australian Dietary Guidelines five food groups: (1) vegetables and legumes/beans; (2) fruit; (3) wholegrain cereal foods and breads; (4) lean meat and poultry, fish, eggs, tofu, seeds and legumes; (5) milk, yoghurt, cheese and alternatives).

Ethical approval to conduct this study was obtained from Hunter New England Human Research Ethics Committee (ref. no. 06/07/26/4.04), University of Newcastle (ref. no. H-2008-0343), and New South Wales (NSW) Department of Education (SERAP 2012277). All trials were prospectively registered with the Australian New Zealand Clinical Trials Registry (ACTRN12613000543785, ACTRN12614001148662, and ACTRN12615001032549).

### Selection of putative mechanisms via the Theoretical Domains Framework

All three trials targeted a range of implementation barriers and enablers outlined by the TDF ––with the hypothesis that addressing these factors would improve guideline implementation. The TDF is an integrative theoretical framework that incorporates 33 theories of behaviour change [22]. The framework includes the following 14 constructs: knowledge; skills; social/professional role and identity; beliefs about capabilities; optimism; beliefs about consequences; reinforcement; intentions; goals; memory, attention and decision processes; environmental context and resources; social influences; behavioural regulation. Detailed definitions of these constructs are reported by Cane et al. [22].

Full details regarding the strategy development process for each trial are described in detail in the respective manuscripts [18–20]. Briefly, in each trial, the development of the implementation strategy was undertaken by a multidisciplinary group including implementation and behavioural scientists, health promotion practitioners, dieticians and those with ‘setting’ (school and childcare service) expertise and followed formative evaluation including literature reviews, quantitative surveys and setting-based observations to identify factors (barriers and enablers). In the SNACS [19] and BMI [20] trial, mapping processes were then undertaken to select implementation strategies that could be employed to address those barriers (or facilitators) to guideline implementation using the TDF strategy selection matrix and further refined following consultations with stakeholders and consideration of project resources and feasibility. The selection of specific Canteen Manager behaviour change techniques in the CAFÉ trial [18] was based on Control Theory [23]. This theory was used to select key behaviour change techniques that would target knowledge gaps and skill barriers.

The constructs targeted by the implementation strategies varied across trials. Thus, for the purposes of testing the most plausible and likely causal mechanisms that would explain how the implementation strategies worked, and limiting the inflation of familywise (type 1) error rate in the aggregate analysis [24], we used a ranking and consensus approach to select four key TDF constructs that converged across all three trials. To do this, we asked a lead investigator from each trial to map and rank all 14 TDF constructs from ‘most likely mechanism’ to ‘least likely mechanism’. All lead investigators participated in each phase of the research project, including the implementation strategy development, stakeholder consultation and implementation strategy delivery. This process was completed independently across trials by three separate trial leads and without any post-hoc knowledge about the intervention effect on any of the TDF constructs. We did this so that the analysis was completely a theory and not data-driven. The independently ranked items were then combined by HL (who was not involved in the planning or conduct of all three trials) in an excel document, and the first four TDF constructs that converged across all three trials were selected for analysis. We did this to avoid the analysts (HL and AH) from having any control over the selection of the TDF domains.

### Measures

We harmonised the outcomes from each trial by computing a binary variable that captured whether a school or childcare centre improved or did not improve in their implementation of nutrition guidelines from baseline [5]. In all trials, canteen managers or childcare service cooks (those primarily responsible for guideline implementation) completed a quantitative TDF survey that assessed all constructs. The survey was adapted from previous TDF instruments to suit the schools and childcare context and was independently validated [25] (Additional file 2). From these surveys, we used measures of four key TDF constructs to represent the primary mediators in this aggregate analysis, including knowledge (5 items), skills (3 items), professional role and identity (3 items), and environmental resources (7 items). We averaged the TDF scores for each construct and computed a standardised score. All TDF constructs and outcomes were measured at the final endpoint of each trial.

### Construction of causal models

**Fig. 1:**
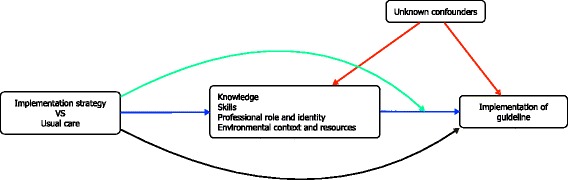
Directed acyclic graph of hypothesised mechanisms. Blue arrows = average causal mediation effect, black arrow = average direct effect, green arrow = intervention-mediator interaction, red arrows = confounding effects. Here we assume that the four mediators are independent of one another

We specified four independent single mediator models with knowledge, skills, professional role and identity, and environmental resources as hypothesised mediators of the intervention effect on policy uptake (directed acyclic graphs shown in Fig. 1). In each model, we assumed that the intervention-mediator and intervention-outcome paths would be unconfounded because of random allocation of treatment [26]. However, for the mediator-outcome effect, we made an explicit assumption that this path could be confounded by unmeasured or unknown confounders. We also assumed that the four mediators were independent of one another [27]. Although it is plausible that the mediators are causally related (for example, better knowledge could increase sense of professional role and identity), given the exploratory nature of this analysis, we decided not to account for intertwined meditators and assumed independence between mediators. Because the causal effect of the mediator on the outcome could depend on the intervention status, we included an intervention-mediator interaction term into the models.

### Statistical analysis

We conducted all analyses in R (The R Foundation for Statistical Computing) using the ‘mediation’ package (version 4.4.6) [28]. We used causal mediation analysis under the counterfactual framework to analyse the aggregate data [13]. For each of the four models, we estimated the average causal mediation effect (ACME), average direct effect (ADE) and the average total effect (ATE). The ACME is the effect of the intervention on the outcome exerted through the hypothesised mediator. The ADE represents the remaining effect of the intervention on the outcome that is not exerted through the selected mediator. Thus, the sum of the ACME and ADE equals the ATE. The proportion of the ATE that is channelled through the mediator (ACME) is termed the proportion mediated.

To analyse each causal model, we fit two regression models: the mediator model and the outcome model. We fit the mediator model using linear regression, specifying intervention allocation and trial ID as independent variables and the mediator as the dependent variable. We then fit the outcome model using binomial probit regression, specifying intervention allocation, trial ID, and mediator as independent variables, and compliance to nutrition policies or guidelines as the dependent variable. In the outcome model, we accounted for the possibility of an intervention-mediator interaction by including the product of intervention allocation and selected mediator into the regression models. To obtain unstandardised point estimates of the quantities of interest (ACME, ADE, ATE), we passed the mediator and outcome models through the mediate command with 1000 bootstrapped simulations [28].

We conducted the primary analysis on complete cases. However, as 16% (*n* = 19) of the primary outcome was missing, we conducted a post hoc sensitivity analysis by using Multiple Imputation by Chained Equations (MICE) [29] to assess the possible impact of missing data. We imputed 20 datasets with 50 iterations and used the bootstrap method to estimate standard errors. We used predictive mean matching to impute continuous variables (TDF domain scores) and logistic regression to impute the binary variable (primary outcome). All four TDF domains, trial, intervention group and the outcome were included in the imputation model. We pooled the estimates and standard errors using Rubin’s rule and calculated 95% CIs as outlined by Carlin et al. [30].

We examined violations in the assumption of normality for the linear regression models (mediator models) via visual inspections of the residual histogram and normal quantile (Q-Q) plots. If normality was violated, we transformed the TDF-construct variable using a BoxCox transformation using the log-likelihood procedure to obtain the optimal power coefficient (lambda) for transformation [31]. We then conducted sensitivity analyses to compare the results from the mediation analysis using transformed TDF-construct variables against the results from the original planned analyses.

#### Sensitivity analysis for sequential ignorability

In a single mediator model, we cannot assume that the mediator-outcome effect is un-confounded because the mediator is not randomised [32]. Thus, to explore the robustness of the ACME to violation of this assumption (sequential ignorability), we conducted sensitivity analyses. The level of confounding due to unknown and unmeasured confounders is represented by the correlation between the residuals from the mediator and outcome regression models (*σ*). We explored if changing the levels of *σ* between − 1 and 1 would influence the ACME and plotted the results.

#### Post hoc power calculation

To gain a general appreciation for the required sample size to detect an indirect effect through the TDF constructs in single mediator models, we used the sample size estimator for joint indirect effects developed by Vittinghoff and Neilands [33]. With a two-sided alpha of 0.05, exposure-mediator error term correlation coefficient of 0, and mediator-outcome error term correlation coefficient of 0.1, a sample of 121 provides 80% power to detect a proportion-mediated of 50%, with meaningful treatment-mediator (*r* = 0.5) and mediator-outcome (*r* = 0.4) effects. This post hoc power calculation provides indication that the pooled analysis would be powered to detect an indirect effect that consists of moderate treatment-mediator and mediator-outcome effects.

## Results

### Descriptive results

**Fig. 2:**
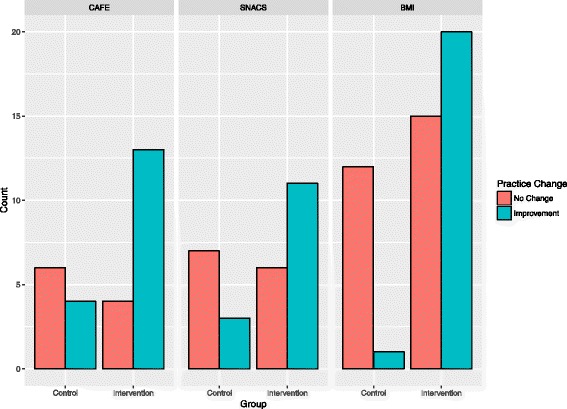
Count of organisations that improved or did not change practice, stratified by trial

In the aggregate dataset (*n* = 121), a total of 42 organisations were randomised to a control group and 79 to an intervention group. The CAFÉ trial contributed 14 control and 23 intervention organisations, SNACS contributed 15 control and 21 intervention organisations and BMI contributed 13 control and 35 intervention organisations. List-wise deletion removed nine (21%) observations from the control group and 10 (11%) observations from the intervention group due to missing data. Nineteen observations were missing for the outcome, and one observation was missing for the TDF domain “Professional Role and Identity” and one from “Environmental context and resources.” In the control group, 8 organisations improved practice and 25 did not. In the intervention group, 44 organisations improved practice and 25 did not. These data stratified by trial are presented in Fig. 2.

### Key findings

**Fig. 3:**
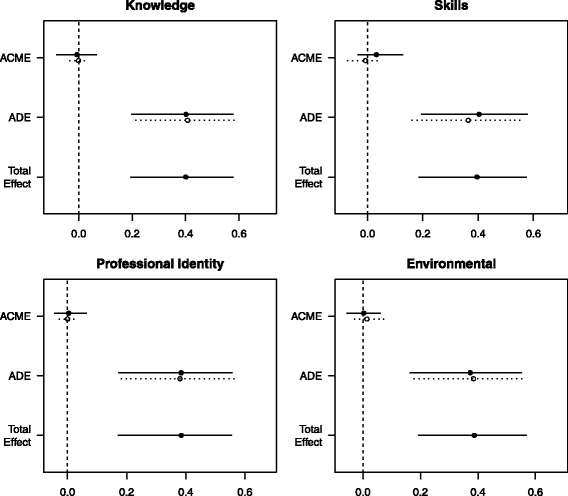
Effect decomposition plots for each mediator model. *ACME* average causal mediation effect, *ADE* average direct effect. Solid dots and lines represent point estimates and 95% confidence limits for the intervention group; the hollow dots and broken lines represent point estimates and confidence limits for the usual care group. The total effect is displayed as an average effect. All effects are reported unstandardized with their 95% confidence intervals

Overall, the intervention group had higher odds of improving adherence to nutritional policy when compared to the control group (odds ratio = 6.64; 95% CI [2.58 to 19.09]). Mediation analyses showed that none of the four TDF constructs explained how the interventions improved policy implementation. This is represented by the small and non-significant ACME and proportion mediated. The analyses showed that most of the intervention effect (ATE) is left unexplained––indicated by significant ADEs that represent unspecified mechanisms. The decompositions of these effects and their precision estimates are shown in Fig. 3.

The regression models for the intervention-mediator path showed that the interventions had a negligible and non-significant effect on all four TDF constructs (second column, Table 2). The regression models for the mediator-outcome path also showed that all four TDF constructs had negligible and non-significant effects on guideline implementation (third column, Table 2). These results indicate that our hypothesised causal mechanism broke down at both the intervention-mediator pathway (action theory) and mediator-outcome pathway (conceptual theory).

**Table 2:** Effect decomposition for four TDF constructs as hypothesised mediators

Mediator	Intervention-mediator effect	Mediator-outcome effect	ATE	ADE	ACME	Proportion mediated (%)
Knowledge	− 0.84 (−5.05 to 3.37)	1.01 (0.96 to 1.06)	0.40 (0.19 to 0.57)*	0.40 (0.20 to 0.58)*	0.00 (− 0.01 to 0.04)	0.00 (− 0.18 to 0.09)
Skills	2.66 (−1.91 to 7.24)	0.99 (0.96 to 1.02)	0.40 (0.19 to 0.57)*	0.38 (0.18 to 0.56)*	0.02 (− 0.02 to 0.08)	0.05 (− 0.08 to 0.21)
Professional role and identity	− 0.88 (− 5.12 to 3.37)	1.00 (0.95 to 1.05)	0.38 (0.17 to 0.55)*	0.38 (0.18 to 0.56)*	0.00 (− 0.03 to 0.03)	0.00 (− 0.09 to 0.09)
Environmental context and resources	− 2.12 (− 6.54 to 2.31)	0.99 (0.96 to 1.03)	0.39 (0.19 to 0.56)*	0.38 (0.18 to 0.56)*	0.00 (− 0.03 to 0.04)	0.00 (− 0.10 to 0.11)

All effects unstandardized with their 95% confidence intervals. The mediator-outcome effects are presented as odds ratios

*ATE* average total effect, *ADE* average direct effect, *ACME* average causal mediation effect

*p = < 0.05

Pooled estimates obtained from the imputed datasets were similar to the results obtained from the complete case analysis. The results are presented in Additional file 3. We observed violations of normality in three linear regression models assessing the following TDF constructs: knowledge, skills, and environmental context and resources. To overcome violations of normality, we used a BoxCox transformation on these three TDF constructs and conducted sensitivity analyses of the mediation models using the transformed variables. We did not observe extreme deviations in the estimates from the sensitivity analyses when compared with the original analyses that used percentage scores of the TDF constructs. The results of the sensitivity analyses are presented in Additional file 4.

**Fig. 4:**
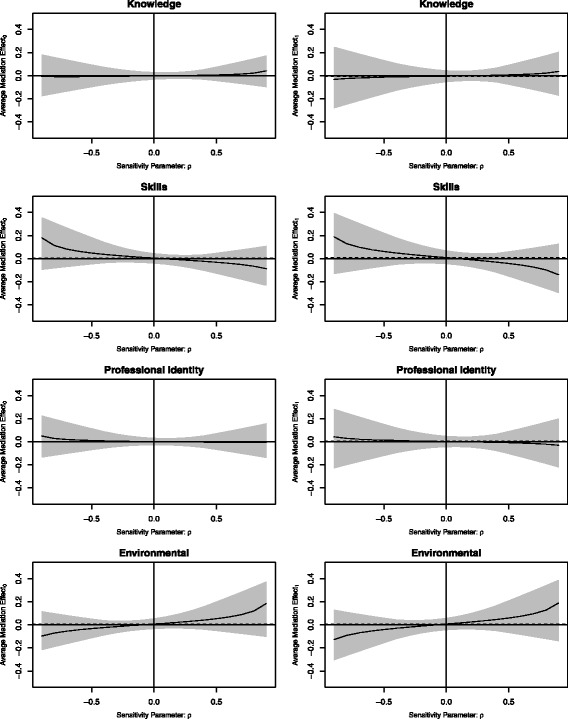
Sensitivity plots. The average mediation effects are plotted as a function of the sensitivity parameter (magnitude of residual confounding). A sensitivity parameter of 0 represents null hypothesised levels of residual confounding and the extremes of − 1 and 1 represent maximum hypothesised levels of residual confounding. Grey zones represent 95% confidence limits of the estimated mediation effect across a range of hypothesised levels of residual confounding

The sensitivity analyses for the sequential ignorability assumption indicated that the ACME estimates were robust. All ACMEs remained stable across low to high levels of unknown and unmeasured confounding (Fig. 4).

## Discussion

This exploratory study sought to identify the mechanisms by which implementation strategies improve nutrition guideline implementation in schools and childcare services. Our aggregate causal mediation analysis found that although the implementation strategies increased adherence to policy and guidelines, none of the theoretically targeted factors (knowledge, skills, professional role and identity, and environmental resources) mediated this effect.

The implementation strategies evaluated in the included trials targeted a range of implementation barriers that were not analysed in this study. It is possible that the interventions were operating via mechanisms that were not captured by the four TDF constructs we explored. For example, previous studies have reported a range of factors associated with successful implementation of healthy eating interventions in childcare––including the support of parents, service management, and the structural availability of time and resources [34]. These factors could have been the active mechanisms of the interventions evaluated in this study but were not captured by the four TDF constructs we explored. Previous work has posited that organisational level change often involves the interplay between many individuals, group and environmental factors [35]. Future research should consider investigating the casual mechanisms that work through system-level factors that could impact the implementation of nutritional policies within childcare services and schools. An ecological systems framework that considers the complex interactions between individuals and their social structures may be appropriate to guide such investigations [36]. Furthermore, the use of the Control Theory in the CAFÉ trial may have caused the intervention to work through mechanisms that were not captured by the TDF.

Another explanation for our findings is that we may have failed to measure the targeted TDF constructs with adequate precision. While the TDF questionnaire used in this analysis has some evidence of validity and reliability [20], it has some limitations. For instance, only one of the three goodness-of-fit statistics from the original confirmatory factor analysis met acceptable criteria [25], which indicates limitations in construct validity. Furthermore, all four constructs of the TDF were negatively skewed for both control and the intervention groups, with most organisations reporting high scores (i.e. low levels of barriers) at follow-up. The skew towards high scores on all four TDF constructs for both groups may reflect possible ceiling effects. If ceiling effects are present, it is possible that our measure was not sensitive enough to detect between group differences, as the measure cannot distinguish respondents at the upper end of the construct [37]. Ceiling effects can attenuate statistical associations, thus resulting in a possible underestimation in the relationship between variables [38, 39]. This may explain why we did not detect a mediating effect through these variables. Future work should seek to make improvements in the current TDF measures to allow for greater sensitivity to detect underlying mechanisms. Possible strategies that may be considered to help reduce the ceiling effects and increase the response variability of the TDF measure could include (i) using more extreme response options at the positive end of the scale, which could help differentiate people who score high. This strategy has been suggested to reduce ceiling effects in other surveys [40, 41], (ii) including survey items that assess respondents’ actual behaviour rather than self-perceived behaviour. For example, rather than asking respondents to indicate whether they are aware of guideline content, it would be more precise to specifically assess the respondents’ actual knowledge of the guideline content. This should help provide a more objective and standardised assessment of barriers.

A strength of this study is the use of pooled data from three relatively large randomised trials in the public health nutrition setting. This was possible through the aggregation of a homogenous collection of trials (same geographic location, similar interventions, similar population and matched target behaviour). This is a key strength as it is often difficult to collect large organisation-level datasets for mechanistic evaluations. Many studies have used the TDF to guide the development of interventions; however, no study has quantitatively tested the TDF constructs as causal mechanisms to refine future implementation strategies [42]. Building an evidence base for the mechanistic role of the TDF constructs will assist in future intervention design and adaptation [7, 12, 42]. Research partnerships between health organisations and clinical trial units should employ similar approaches to conduct mechanism-focused implementation studies. Planning and executing a concerted set of trials that assess similar implementation mechanisms could yield robust evidence for how implementation strategies work or do not work. These techniques can and should be applied across various settings for better implementation of preventive and healthcare strategies.

Our findings should also be interpreted in the context of its limitations. We were unable to adjust for any confounders of the mediator-outcome effect. However, our sensitivity analysis indicated that our estimations of the mediation effect would remain stable even at high levels of residual confounding. We may have measured the TDF constructs with error and poor precision. It is possible that the questionnaire used to assess the TDF constructs was limited in its construct validity and displayed ceiling effects. The temporal precedence between the mediator, and outcome is unclear in our analyses. Future work should aim to measure the mediator prior to the outcome and assess the possibility of reverse causation. The trial participants may have felt under pressure to report excellent knowledge and skills after training (social desirability bias). However, given that we did not detect between group differences on knowledge and skills, the likelihood of this bias is low. Lastly, 19 (16%) organisations were lost to follow-up, and this may have induced bias if the missingness mechanism was not at random.

## Conclusions

Understanding the casual mechanisms of a complex implementation strategy can inform the development and adaptation of future strategies, as well as provide an opportunity to understand and improve the theoretical underpinnings of implementation science. As the first study of its kind in this setting, an aggregated causal mediation analysis of three complex intervention trials that aimed to improve the implementation of nutritional guidelines across Australian primary schools and childcare services showed that none of the four hypothesised TDF constructs were meaningful mediators. Future research should employ similar methods and techniques to explore the mechanistic role of other TDF constructs and evaluate system-level mechanisms informed by an ecological framework.

## Additional files

Additional file 1: Cochrane Risk of Bias Assessment. (DOCX 110 kb)

Additional file 2: School survey questions and modified questions. (DOCX 35 kb)

Additional file 3: Sensitivity analysis using multiple imputation to replace missing values. (DOCX 17 kb)

Additional file 4: Sensitivity analysis using transformed TDF variables. (DOCX 60 kb)

## Abbreviations

ACME: Average causal mediation effect
ADE: Average direct effect
ATE: Average total effect
TDF: Theoretical Domains Framework
