# Supplementary material for: Mechanisms of implementing public health interventions: a pooled causal mediation analysis of randomised trials
Source: Implement Sci. 2018 Mar 12;13:42. doi: 10.1186/s13012-018-0734-9 (PMC5848564; doi:10.1186/s13012-018-0734-9)
Supplement: Supplementary file 1 — Cochrane Risk of Bias Assessment. (DOCX 110 kb) [file 13012_2018_734_MOESM1_ESM.docx]

**S1: Cochrane Risk of Bias Assessment**

***Nathan N, Yoong SL, Sutherland R, et al. Effectiveness of a multicomponent intervention to enhance implementation of a healthy canteen policy in Australian primary schools: a randomised controlled trial. Int J Behav Nutr Phys Act 2016;13:106.***

| **Bias** | **Authors' judgement** | **Support for judgement** |
| --- | --- | --- |
| Random sequence generation (selection bias) | Low risk | Randomised controlled trial. The random sequence was generated using a computerised random number function in Microsoft Excel. |
| Allocation concealment (selection bias) | Unclear risk | Group allocation was concealed from staff involved in school recruitment however there is no information about how allocation was concealed. |
| Blinding of participants and personnel (performance bias) | High risk | Outcome group: Schools were not blinded to group allocation and therefore at high risk of performance bias. |
| Blinding of outcome assessment (detection bias) | Low risk | Outcome group: Dietitians conducting menu assessments were blind to group allocation. |
| Incomplete outcome data (attrition bias) | Low risk | Outcome group: Only one school was lost to follow-up. |
| Selective reporting (reporting bias) | Low risk | The trial was prospectively registered with the Australian New Zealand Clinical Trials Registry (ACTRN12614001148662).  All predetermined outcomes were reported. |
| Recruitment to cluster | Unclear risk |  |
| Baseline imbalance | Unclear risk |  |
| Loss of cluster | Unclear risk |  |
| Incorrect analysis | Unclear risk |  |
| Contamination | Unclear risk |  |
| Compatibility with individually randomised RCTs | Unclear risk |  |
| Potential confounding | Unclear risk |  |

***Yoong SL, Nathan N, Wolfenden L, et al. CAFÉ: a multicomponent audit and feedback intervention to improve implementation of healthy food policy in primary school canteens: a randomised controlled trial. Int J Behav Nutr Phys Act 2016;13:126.***

| **Bias** | **Authors' judgement** | **Support for judgement** |
| --- | --- | --- |
| Random sequence generation (selection bias) | Low risk | Randomised controlled trial. The random sequence was produced using a computer-generated randomisation schedule in Microsoft Excel. |
| Allocation concealment (selection bias) | Unclear risk | There is no information provided about allocation concealment and therefore it is unclear if allocation was concealed. |
| Blinding of participants and personnel (performance bias) | High risk | Outcome group: Due to the nature of the intervention schools could not be blinded to group allocation and therefore at high risk of performance bias. |
| Blinding of outcome assessment (detection bias) | Low risk | Outcome group: Menu audits were undertaken by dietitians blinded to group allocation. |
| Incomplete outcome data (attrition bias) | Low risk | Outcome group: 19 of the 72 (26%) schools did not provide menus at follow-up. There were no significant differences in baseline characteristics among schools that did and did not provide follow-up data. |
| Selective reporting (reporting bias) | Low risk | There were no unreported implementation outcomes according to those planned in the published protocol. |
| Recruitment to cluster | Unclear risk |  |
| Baseline imbalance | Unclear risk |  |
| Loss of cluster | Unclear risk |  |
| Incorrect analysis | Unclear risk |  |
| Contamination | Unclear risk |  |
| Compatibility with individually randomised RCTs | Unclear risk |  |
| Potential confounding | Unclear risk |  |

***Seward K, Wolfenden L, Finch M, Wiggers J, Wyse R, Jones J, Gillham K, Yoong SL. Multistrategy childcare-based intervention to improve compliance with nutrition guidelines versus usual care in long day care services: a study protocol for a randomised controlled trial. BMJ open. 2016;6(6):10786. doi:10.1136/bmjopen-2015-010786***

| **Bias** | **Authors' judgement** | **Support for judgement** |
| --- | --- | --- |
| Random sequence generation (selection bias) | Low risk | Randomised controlled trial. The random sequence was produced in 1:1 ratio via block randomisation using a random number function in SAS statistical software. Block size ranged between 2 and 6. |
| Allocation concealment (selection bias) | Unclear risk | Author mentioned allocation of services would be undertaken by an experienced research assistant. There is no information provided about allocation concealment and therefore it is unclear if allocation was concealed. |
| Blinding of participants and personnel (performance bias) | High risk | Due to the nature of the intervention childcare service staff were aware of their group allocation and therefore at high risk of performance bias. |
| Blinding of outcome assessment (detection bias) | Low risk | Menu assessments were undertaken by dietitians blinded to group allocation at baseline and at 6 months follow up. All outcome data collectors were blinded to group allocation. |
| Incomplete outcome data (attrition bias) | Low risk | Only one childcare service (intervention group) did not complete follow up data collection, however reasons and characteristics were not reported. Intent-to-treat (multiple imputation) analysis was used for missing data. |
| Selective reporting (reporting bias) | Low risk | The trial was prospectively registered with the Australian New Zealand Clinical Trials Registry (ACTRN12615001032549). All predetermined outcomes were reported. |
| Recruitment to cluster | Unclear risk |  |
| Baseline imbalance | Unclear risk |  |
| Loss of cluster | Unclear risk |  |
| Incorrect analysis | Unclear risk |  |
| Contamination | Unclear risk |  |
| Compatibility with individually randomised RCTs | Unclear risk |  |
| Potential confounding | Unclear risk |  |
