# Supplementary material for: Mechanisms of implementing public health interventions: a pooled causal mediation analysis of randomised trials
Source: Implement Sci. 2018 Mar 12;13:42. doi: 10.1186/s13012-018-0734-9 (PMC5848564; doi:10.1186/s13012-018-0734-9)
Supplement: Supplementary file 2 — School survey questions and modified questions. (DOCX 35 kb) [file 13012_2018_734_MOESM2_ESM.docx]

**S2 Appendix. School survey questions and modified questions.**

| TDF Domain | Statement / Survey question |
| --- | --- |
| Knowledge | I am aware of the content of the FT@S guidelines. |
|  | I am aware of the objectives of the FT@S guidelines. |
|  | I know how to plan a menu according to the FT@S guidelines. |
| Skills | I have received training regarding how to plan a menu according to the FT@S guidelines. |
|  | I have the skills needed to develop a canteen menu according to the FT@S guidelines. |
|  | I am confident I can determine if a product falls into the RED category according to the FT@S guidelines. |
|  | I am confident I can determine if a product falls into the GREEN category according to the FT@S guidelines. |
| Social/Professional role & identity | Planning a menu according to the FT@S guidelines, is part of my role. |
|  | It is my responsibility to plan a menu according to the FT@S guidelines. |
|  | Planning a menu according to the FT@S guidelines is consistent with other aspects of my job. |
| Beliefs about capabilities | I am confident that I can plan a menu according to the FT@S guidelines. |
|  | I have personal control over planning a menu according to the FT@S guidelines. |
|  | For me, planning a menu according to the FT@S guidelines, is easy. |
| Optimism | In uncertain times, when planning a menu according to the FT@S guidelines, I usually expect that things will work out ok. |
|  | When I plan a menu according to the FT@S guidelines, I feel optimistic about my job in the future. |
|  | I do not expect anything will prevent me from planning a menu according to the FT@S guidelines. |
| Beliefs about consequences | I believe planning a menu according to the FT@S guidelines will lead to benefits for the children who attend this school. |
|  | In my view, planning a menu according to the FT@S guidelines is worthwhile. |
|  | I believe planning a menu according to the FT@S guidelines will benefit public health. |
| Reinforcement | When I plan a menu according to the FT@S guidelines I get recognition from my colleagues. |
|  | I get recognition from management at the school where I work when I plan a menu according to the FT@S guidelines. |
|  | When I plan a menu according to the FT@S guidelines I get recognition from those who it impacts. |
| Intentions | I intend to plan a menu according to the FT@S guidelines at every menu review. |
|  | I will definitely plan a menu according to the FT@S guidelines, at every menu review. |
|  | I have a strong intention to plan a menu according to the FT@S guidelines, at every menu review. |
| Goals | Compared to my other tasks, planning a menu according to the FT@S guidelines is a higher priority on my agenda. |
|  | Compared to my other tasks, planning a menu according to the FT@S guidelines is an urgent item on my agenda. |
|  | I set achievable short-term goals when planning a menu according to the FT@S guidelines. |
|  | I have clear long-term goals related to planning a menu according to the FT@S guidelines. |
| Memory, attention & decision processes | Planning a menu according to the FT@S guidelines is something I do automatically. |
|  | Planning a menu according to the FT@S guidelines is something I forget. |
| Environmental context & resources | I have support from the Principal of the school to plan a menu according to the FT@S guidelines. |
|  | I have support from the parents of the school to plan a menu according to the FT@S guidelines. |
|  | In the school I work, all necessary resources are available to Plan a menu according to the FT@S guidelines. |
|  | Planning a menu according to the FT@S guidelines aligns with the goals, objectives and/or philosophy of the school where I work. |
| Social Influences | People who are important to me think that I should plan a menu according to the FT@S guidelines. |
|  | People whose opinion I value would approve of me planning a menu according to the FT@S guidelines at every menu review. |
|  | Other staff/volunteers whom I work with are willing to listen to my problems when Planning a menu according to the FT@S guidelines at every menu review. |
|  | Children at the school where I plan a menu according to the FT@S guidelines are receptive. |
| Emotion | I feel comfortable planning a menu according to the FT@S guidelines. |
|  | Planning a menu according to the FT@S guidelines makes me feel good. |
|  | I am able to plan a menu according to the FT@S guidelines without feeling anxious. |
| Behavioural regulation | Planning a menu according to the FT@S guidelines is simple. |
|  | Planning a menu according to the FT@S guidelines takes little time to deliver. |
|  | It is possible to adapt how I plan a menu according to the FT@S guidelines to meet my needs as a canteen manager. |
|  | Planning a menu according to the FT@S guidelines is compatible with other aspects of my job. |

**Canteen Manager CATI**

| **Domain** | **Paper 1 (32 items)** | **Paper 2 (93 items)** | **Item #** | **Proposed Item** | **Question for Canteen Managers** |
| --- | --- | --- | --- | --- | --- |
| **D1. Knowledge**  **(3 items)** | I know the content and objectives of [innovation/guideline] | Objectives of [PA intervention] and my role in this are clearly defined for me. | **10** | **I am aware of the content of the** [insert name of recommendations, protocol, guidelines] | **I am aware of the content of the** FT@S guidelines. |
|  |  |  | **11** | **I am aware of the objectives of the** [insert name of recommendations, protocol, guidelines] | **I am aware of the objectives of the** FT@S guidelines. |
|  | I am aware of the content and objectives of [innovation/guideline] | I know how to deliver [PA intervention] following the guidelines. | **12** | **I know how to [insert action related to program, intervention, innovation or guidelines] according to the [insert name of recommendations, protocol, guidelines]** | **I know how to plan a menu according to the FT@S guidelines.** |

| **Domain** | **Paper 1 (32 items)** | **Paper 2 (79 items)** | **Item #** | **Proposed Item** | **Proposed question for Canteen Managers** |
| --- | --- | --- | --- | --- | --- |
| **D2. Skills**  **(4 items)** | I have been trained how to [A] in [C, T] with [Ta] | I have been trained in delivering [PA intervention] following the guidelines. | **13** | **I have received training regarding how to** [insert action related to program, intervention, innovation or guidelines] **according to the** [insert name of recommendations, protocol, guidelines] | **I have received training regarding how to** plan a menu **according to the** FT@S guidelines. |
|  | I have the skills to [A] in [C, T] with [Ta] | I have the skills to deliver [PA intervention] following the guidelines. | **14** | **I have the skills needed to** [insert action related to program, intervention, innovation or guidelines] **according to the** [insert name of recommendations, protocol, guidelines] | **I have the skills needed to** plan a menu **according to the** FT@S guidelines. |
|  |  |  | **15** |  | I am confident I can determine if a product falls into the RED category according to the FT@S guidelines. |
|  |  |  | **16** |  | I am confident I can determine if a product falls into the GREEN category according to the FT@S guidelines. |

| **Domain** | **Paper 1 (32 items)** | **Paper 2 (79 items)** | **Item #** | **Proposed Item** | **Proposed question for Canteen Managers** |
| --- | --- | --- | --- | --- | --- |
| **D3. Social/professional role and identity**  **(3 items)** | [A] in [C, T] with [Ta] is part of my work as a [profession] | Delivering [PA intervention] following the guidelines  is part of my work as a PT. | **33** | [Insert action related to program, intervention, innovation or guidelines] **according to the** [insert name of recommendations, protocol, guidelines], **is part of my role** | Planning a menu **according to the** FT@S guidelines, **is part of my role.** |
|  | It is my responsibility as a [profession] to [A] in [C, T] with [Ta] | It is my responsibility as a PT to deliver [PA intervention] following the guidelines. | **34** | **It is my responsibility to** [insert action related to program, intervention, innovation or guidelines] **according to the** [insert name of recommendations, protocol, guidelines] | **It is my responsibility to** plan a menu **according to** the FT@S guidelines. |
|  | Doing [A] in [C, T] with [Ta] is consistent with my [profession] |  | **35** | [Insert action related to program, intervention, innovation or guidelines] **according to the** [insert name of recommendations, protocol, guidelines], **is consistent with other aspects of my job** | Planning a menu **according to** the FT@S guidelines **is consistent with other aspects of my job.** |

| **Domain** | **Paper 1 (32 items)** | **Paper 2 (79 items)** | **Item #** | **Proposed Item** | **Proposed question for Canteen Managers** |
| --- | --- | --- | --- | --- | --- |
| **D4. Beliefs about capabilities**  **(3 items)** | I am confident that if I wanted I could [A] in [C, T] with [Ta] | I am confident that I can deliver [PA intervention] following the guidelines. | **27** | **I am confident that I can** [insert action related to program, intervention, innovation or guidelines] **according to the** [insert name of recommendations, protocol, guidelines] | **I am confident that I can** plan a menu **according to the** FT@S guidelines. |
|  |  | I have control over delivering [PA intervention] following the guidelines. | **28** | **I have personal control over** [insert action related to program, intervention, innovation or guidelines] **according to the** [insert name of recommendations, protocol, guidelines] | **I have personal control over** planning a menu **according to the** FT@S guidelines. |
|  |  | For me, delivering [PA intervention] following the guidelines is (very difficult – very easy). | **29** | **For me,** [insert action related to program, intervention, innovation or guidelines] **according to the** [insert name of recommendations, protocol, guidelines]**, is easy** | **For me,** planning a menu **according to the** FT@S guidelines **is easy.** |
| **D5. Optimism**  **(3 items)** | With regard to [A] in [C, T] with [Ta] in uncertain times, I usually expect the best | In my work as a PT, in uncertain times, I usually expect the best. |  | Even when I feel uncertain about my ability to [insert action related to program, intervention, innovation or guidelines] according to the [insert name of recommendations, protocol, guidelines], I usually expect that things will work out okay | **In uncertain times, when** planning a menu **according to the** FT@S guidelines**, I usually expect that things will work out okay.** |
|  | With regard to [A] in [C, T] with [Ta] I’m always optimistic about the future | In my work as a PT, I’m always optimistic about the future. |  | **When I** [insert action related to program, intervention, innovation or guidelines] **according to the** [insert name of recommendations, protocol, guidelines]**, I feel optimistic about my job in the future** | **When I** plan a menu **according to the** FT@S guidelines**, I feel optimistic about my job in the future.** |
|  |  |  |  | **I do not expect anything will prevent me from** [insert action related to program, intervention, innovation or guidelines] **according to the** [insert name of recommendations, protocol, guidelines] | **I do not expect anything will prevent me from** planning a menu **according to the** FT@S guidelines |
| **D6. Beliefs about consequences**  **(3 items)** |  |  |  | **I believe** [insert action related to program, intervention, innovation or guidelines] **according to the** [insert name of recommendations, protocol, guidelines]**, will lead to benefits for the** [participants, clients, patients, individuals, children] | **I believe** planning a menu **according to the** FT@S guidelines **will lead to benefits for the** children who attend this school. |
|  |  | For me, delivering [PA intervention] following the guidelines is  (not worthwhile at all – very worthwhile). |  |  | **In my view,** planning a menu **according to** the FT@S guidelines **is worthwhile** |
|  | If I [A] in [C, T] with [Ta] it will benefit public health |  |  | **I believe** [insert action related to program, intervention, innovation or guidelines] **according to the** [insert name of recommendations, protocol, guidelines], **will benefit public health.** | **I believe** planning a menu **according to the** FT@S guidelines, **will benefit public health.**  **Interviewer note: define public health ‘ie health of the whole population, obesity prevention’** |
| **D7. Reinforcement**  **(3 items)** |  | When I deliver [PA intervention] following the guidelines,  I get recognition from the work context. |  | **When I** [insert action related to program, intervention, innovation or guidelines] **according to the** [insert name of recommendations, protocol, guidelines]**, I get recognition from my colleagues** | **When I** plan a menu **according to the** FT@S guidelines **I get recognition from my colleagues.** |
|  |  |  |  | **I get recognition from management at the organisation where I work, when I** [insert action related to program, intervention, innovation or guidelines] **according to the** [insert name of recommendations, protocol, guidelines] | **I get recognition from management at the school where I work, when I** plan a menu **according to the** FT@S guidelines. |
|  |  |  |  | **When I** [insert action related to program, intervention, innovation or guidelines] **according to the** [insert name of recommendations, protocol, guidelines]**, I get recognition from those who it impacts** | **When I** plan a menu **according to the** FT@S guidelines**, I get recognition from those who it impacts - or should it be from students and/or parents??** |

| **Domain** | **Paper 1 (32 items)** | **Paper 2 (79 items)** | **Item #** | **Proposed Item** | **Proposed question for Canteen Managers** |
| --- | --- | --- | --- | --- | --- |
| **D8. Intentions**  **(3 items)** | For how many of the next 10 [Ta] do you intend to [A] in [C]? | I intend to deliver [PA intervention] following the guidelines  in the next three months. | **36** | **I intend to** [insert action related to program, intervention, innovation or guidelines] **according to** the [insert name of recommendations, protocol, guidelines], **at** [each/every time relevant to action] | **I intend to** plan a menu **according to** the FT@S guidelines **at** every menu review |
|  | I will definitely [A] in [C] with [Ta] in the next [T] | I will definitely deliver [PA intervention] following the  guidelines in the next three months. | **37** | **I will definitely** [insert action related to program, intervention, innovation or guidelines] **according to** the [insert name of recommendations, protocol, guidelines], **at** [each/every time relevant to action] | **I will definitely** plan a menu **according to** the FT@S guidelines, **at** every menu review |
|  | How strong is your intention to [A] with [Ta] in [C] in the next [T]? | How strong is your intention to deliver [PA intervention]  following the guidelines in the next three months? | **38** | **I have a strong intention to** [insert action related to program, intervention, innovation or guidelines] **according to** the [insert name of recommendations, protocol, guidelines], **at** [each/every time relevant to action] | **I have a strong intention to** plan a menu **according to** the FT@S guidelines, **at** every menu review |

| **Domain** | **Paper 1 (32 items)** | **Paper 2 (79 items)** | **Item #** | **Proposed Item** | **Proposed question for Canteen Managers** |
| --- | --- | --- | --- | --- | --- |
| **D9. Goals**  **(4 items)** | **discriminant content validity of the items measuring these domains was not demonstrated | How often is working on something else on your agenda a  higher priority than delivering [PA intervention] following  the guidelines? | **19** | **Compared to my other tasks,** [insert action related to program, intervention, innovation or guidelines] **according to the** [insert name of recommendations, protocol, guidelines], **is a higher priority on my agenda** | **Compared to my other tasks,** planning a menu **according to the** FT@S guidelines **is a higher priority on my agenda** |
|  |  | How often is working on something else on your agenda  more urgent than delivering [PA intervention] following  the guidelines? | **20** | **Compared to my other tasks,** [insert action related to program, intervention, innovation or guidelines] **according to the** [insert name of recommendations, protocol, guidelines], **is an urgent item on my agenda** | **Compared to my other tasks,** planning a menu **according to the** FT@S guidelines **is an urgent item on my agenda** |
|  |  |  | **21** | **I have clear long-term goals related to** [insert action related to program, intervention, innovation or guidelines] **according to the** [insert name of recommendations, protocol, guidelines] | **I have clear long-term goals related to** planning a menu **according to the** FT@S guidelines. |
|  |  |  | **22** | **I set achievable short-term goals when** [insert action related to program, intervention, innovation or guidelines] **according to the** [insert name of recommendations, protocol, guidelines] | **I set achievable short-term goals when** planning a menu **according to the** FT@S guidelines. |

| **Domain** | **Paper 1 (32 items)** | **Paper 2 (79 items)** | **Item #** | **Proposed Item** | **Proposed question for Canteen Managers** |
| --- | --- | --- | --- | --- | --- |
| **D10. Memory, attention, and decision processes**  **(2 items)** | How often do you forget [A] in [C, T] with [Ta]? | Delivering [PA intervention] following the guidelines is  something I do automatically. | **17** | [Insert action related to program, intervention, innovation or guidelines] **according to the** [insert name of recommendations, protocol, guidelines] **is something I do automatically** | Planning a menu **according to the** FT@S guidelines **is something I do automatically.** |
|  |  |  |  |  |  |
|  |  | Delivering [PA intervention] following the guidelines is  something I often forget. | **18** | [Insert action related to program, intervention, innovation or guidelines] **according to the** [insert name of recommendations, protocol, guidelines] **is something I forget** | Planning a menu **according to the** FT@S guidelines **is something I forget.** |

| **Domain** | **Paper 1 (32 items)** | **Paper 2 (79 items)** | **Item #** | **Proposed Item** | **Proposed question for Canteen Managers** |
| --- | --- | --- | --- | --- | --- |
| **D11. Environmental context and resources**  **(4 items)** | Within the socio-political context there is sufficient financial support (e.g., from local authorities, insurance  companies, the government) for [innovation/guideline] | In the organization I work, all necessary resources are  available to deliver [PA intervention]. | 44 | **In the organisation I work, all necessary resources are available to** [Insert action related to program, intervention, innovation or guidelines] **according to the** [insert name of recommendations, protocol, guidelines]] | **In the school I work, all necessary resources are available to** Plan a menu **according to the** FT@S guidelines. |
|  | Within the socio-political context there are good networks between parties involved in [innovation/guideline] | I can count on support from the management of the  organization I work in, when things get tough guidelines. | 39 | **I have support from the management of the organisation to** [Insert action related to program, intervention, innovation or guidelines] **according to the** [insert name of recommendations, protocol, guidelines] | **I have support from the Principal of the school to** plan a menu **according to the** FT@S guidelines. |
|  |  |  | 40 |  | **I have support from the parents of the school to** plan a menu **according to the** FT@S guidelines**.** |
|  |  |  | 45 | [Insert action related to program, intervention, innovation or guidelines] **according to the** [insert name of recommendations, protocol, guidelines] **aligns with the goals, objectives and/or philosophy of the organisation where I work** | Planning a menu **according to the** FT@S guidelines **aligns with the goals, objectives and/or philosophy of the school where I work.** |

| **Domain** | **Paper 1 (32 items)** | **Paper 2 (79 items)** | **Item #** | **Proposed Item** | **Proposed question for Canteen Managers** |
| --- | --- | --- | --- | --- | --- |
| **D12. Social influences**  **(3 items)** | Most people who are important to me think that I should [A] in [C, T] with [Ta] | Most people who are important to me think that  I should deliver [PA intervention] following the guidelines. | 41 | **People who are important to me think that I should** [Insert action related to program, intervention, innovation or guidelines] **according to the** [insert name of recommendations, protocol, guidelines] | **People who are important to me think that I should** plan a menu **according to the** FT@S guidelines. |
|  | Most people whose opinion I value would approve me of [A] in [C, T] with [Ta] | Professionals with whom I deliver [PA intervention]  think I should deliver [PA] intervention] following the guidelines. | 42 | **People whose opinion I value would approve of me** [Insert action related to program, intervention, innovation or guidelines] **according to the** [insert name of recommendations, protocol, guidelines] **at** [each/every time relevant to action] | **People whose opinion I value would approve of me** planning a menu **according to the** FT@S guidelines **at** every menu review |
|  |  | Professionals with whom I deliver [PA intervention]  are willing to listen to my problems with delivering  [PA intervention] following the guidelines. | 43 | **Colleagues whom I work with are willing to listen to my problems when** [Insert action related to program, intervention, innovation or guidelines] **according to the** [insert name of recommendations, protocol, guidelines] **at** [each/every time relevant to action] | **Other canteen staff/volunteers whom I work with are willing to listen to my problems when** Planning a menu **according to the** FT@S guidelines **at** every menu review |

| **Domain** | **Paper 1 (32 items)** | **Paper 2 (93 items)** | **Item #** | **Proposed Item** | **Proposed question for Canteen Manager** |
| --- | --- | --- | --- | --- | --- |
| **D.13 Emotion**  **(3 items)** |  | When I work with [PA intervention] I feel comfortable. | 30 | **I feel comfortable** [insert action related to program, intervention, innovation or guidelines] **according to the** [insert name of recommendations, protocol, guidelines] | **I feel comfortable** planning a menu **according to the** FT@S guidelines. |
|  |  | When I work with [PA intervention] I feel cheerful. | 31 | [insert action related to program, intervention, innovation or guidelines] **according to the** [insert name of recommendations, protocol, guidelines], **makes me feel good** | Planning a menu **according to the** FT@S guidelines **makes me feel good.** |
|  |  | When I work with [PA intervention] I feel nervous. | 32 | I am able to [insert action related to program, intervention, innovation or guidelines] according to the [insert name of recommendations, protocol, guidelines], without feeling nervous or anxious | I am able to plan a menu according to the FT@S guidelines without feeling anxious. |

| **Domain** | **Paper 1 (32 items)** | **Paper 2 (79 items)** | **Item #** | **Proposed Item** | **Proposed question for Canteen Manager** |
| --- | --- | --- | --- | --- | --- |
| **D.14 Behavioural Regulation**  **(4 items)** |  | It is possible to tailor [PA intervention] to professionals’ needs? | 25 | **It is possible to adapt how I** [insert action related to program, intervention, innovation or guidelines] **according to the** [insert name of recommendations, protocol, guidelines] **to meet the my needs as a** [insert role] | **It is possible to adapt how I** plan a menu **according to the** FT@S guidelines **to meet the my needs as a** canteen manager. |
|  |  | [PA intervention] costs little time to deliver. | 24 | [insert action related to program, intervention, innovation or guidelines] **according to the** [insert name of recommendations, protocol, guidelines] **takes little time to deliver** | Planning a menu **according to the** FT@S guidelines **takes little time to deliver.** |
|  |  | [PA intervention] is compatible with daily practice. | 26 | [insert action related to program, intervention, innovation or guidelines] **according to the** [insert name of recommendations, protocol, guidelines] **is compatible with other aspects of my job** | Planning a menu **according to the** FT@S guidelines **is compatible with other aspects of my job.** |
|  |  | [PA intervention] is simple to deliver. | 23 | [insert action related to program, intervention, innovation or guidelines] **according to the** [insert name of recommendations, protocol, guidelines] **is simple** | Planning a menu **according to the** FT@S guidelines **is simple.** |

**References:**

**Paper 1:** Huijg et al.: Discriminant content validity of a theoretical domains framework questionnaire for use in implementation research. Implementation Science 2014 9:11.

**Paper 2:** Huijg et al.: Measuring determinants of implementation behavior: psychometric properties of a questionnaire based on the theoretical domains framework. Implementation Science 2014 9:33.

**Domain definitions**

| **Domain** | **Constructs** |
| --- | --- |
| Knowledge | Knowledge (including knowledge of condition /scientific rationale), Procedural knowledge,  Knowledge of task environment |
| Skills | Skills,  Skills development,  Competence,  Ability,  Interpersonal skills,  Practice,  Skill assessment  Coping strategies |
| Professional role and identity | Professional identity,  Professional role,  Social identity,  Identity,  Professional boundaries,  Professional confidence,  Group identity,  Leadership,  Organisational commitment |
| Beliefs about capabilities | Self‐confidence,  Perceived competence,  Self‐efficacy,  Perceived behavioural control, Beliefs,  Self‐esteem,  Empowerment,  Professional confidence |
| Optimism | Optimism,  Pessimism,  Unrealistic optimism,  Identity |
| Beliefs about consequences | Beliefs,  Outcome expectancies, Characteristics of outcome expectancies,  Anticipated regret,  Consequents |
| Reinforcement | Rewards (proximal / distal, valued / not valued, probable / improbable), Incentives,  Punishment,  Consequents,  Reinforcement,  Contingencies,  Sanctions |
| Intentions | Stability of intentions,  Stages of change model, Transtheoretical model and stages of change |
| Goals | Goals (distal / proximal),  Goal priority,  Goal / target setting,  Goals (autonomous /  controlled),  Action planning,  Implementation intention |
| Memory, attention and decision processes | Memory,  Attention,  Attention control,  Decision making,  Cognitive overload / tiredness |
| Environmental context and resources | Environmental stressors,  Resources / material resources, Organisational culture /climate,  Salient events / critical incidents, Person x environment interaction, Barriers and facilitators |
| Social influences | Social pressure,  Social norms,  Group conformity,  Social comparisons,  Group norms,  Social support,  Power,  Intergroup conflict,  Alienation,  Group identity,  Modelling |
| Emotions | Fear,  Anxiety,  Affect,  Stress,  Depression,  Positive / negative affect,  Burn‐out |
| Behavioural regulation | Self‐monitoring,  Breaking habit,  Action planning |
