# Supplementary material for: Mechanisms of implementing public health interventions: a pooled causal mediation analysis of randomised trials
Source: Implement Sci. 2018 Mar 12;13:42. doi: 10.1186/s13012-018-0734-9 (PMC5848564; doi:10.1186/s13012-018-0734-9)
Supplement: Supplementary file 3 — Sensitivity analysis using multiple imputation to replace missing values. (DOCX 17 kb) [file 13012_2018_734_MOESM3_ESM.docx]

**Appendix 3. Sensitivity analysis using multiple imputation to replace missing values**

| **Mediator** | **Intervention-mediator effect** | **Mediator-outcome effect** | **ATE** | **ADE** | **ACME** | **Proportion mediated (%)** |
| --- | --- | --- | --- | --- | --- | --- |
|  |  |  |  |  |  |  |
| Knowledge | -0.84 (-5.05, 3.37) | 1.02 (0.97, 1.07) | 0.39 (0.20, 0.58) | 0.40 (0.21, 0.58) | -0.01 (-0.05, 0.03) | -0.02 (-0.14, 0.10) |
|  |  |  |  |  |  |  |
| Skills | 2.66 (-1.91, 7.24) | 0.99 (0.96, 1.02) | 0.37 (0.19, 0.56) | 0.36 (0.18, 0.55) | 0.01 (-0.03, 0.05) | 0.03 (-0.07, 0.13) |
|  |  |  |  |  |  |  |
| Professional role and identity | -0.65 (-4.94, 3.63) | 1.01 (0.97, 1.06) | 0.40 (0.21, 0.58) | 0.40 (0.22, 0.58) | -0.00 (-0.03, 0.03) | -0.01 (-0.10, 0.08) |
|  |  |  |  |  |  |  |
| Environmental context and resources | -1.89 (-6.39, 2.62) | 0.99 (0.96, 1.02) | 0.40 (0.22, 0.59) | 0.40 (0.21, 0.58) | 0.00 (-0.03, 0.04) | 0.01 (-0.08, 0.10) |

All effects unstandardized with their 95% confidence intervals. The mediator-outcome effects are presented as odds ratios.

ATE = average treatment effect; ADE = average direct effect; ACME = average causal mediation effect
