# Supplementary material for: Mechanisms of implementing public health interventions: a pooled causal mediation analysis of randomised trials
Source: Implement Sci. 2018 Mar 12;13:42. doi: 10.1186/s13012-018-0734-9 (PMC5848564; doi:10.1186/s13012-018-0734-9)
Supplement: Supplementary file 4 — Sensitivity analysis using transformed TDF variables. (DOCX 60 kb) [file 13012_2018_734_MOESM4_ESM.docx]

**S4. Sensitivity analysis using transformed TDF variables.**

| **Mediator** | **Intervention-mediator effect** | **Mediator-outcome effect** | **ATE** | **ADE** | **ACME** | **Proportion mediated (%)** |
| --- | --- | --- | --- | --- | --- | --- |
|  |  |  |  |  |  |  |
| Knowledge | -505.20 (-3506.23 to 2495.79) | 1.00 (1.00 to 1.00) | 0.40 (0.19 to 0.58)* | 0.40 (0.21 to 0.58)* | -0.00 (-0.05 to 0.04) | -0.00 (-0.17 to 0.09) |
|  |  |  |  |  |  |  |
| Skills | 211.90 (-319.92 to 743.71) | 1.00 (1.00 to 1.00) | 0.40 (0.19 to 0.57)* | 0.38 (0.18 to 0.56)* | 0.01 (-0.02 to 0.07) | 0.03 (-0.07 to 0.19) |
|  |  |  |  |  |  |  |
| Professional role and identity | -0.88 (-5.12 to 3.37) | 1.00 (0.95 to 1.05) | 0.38 (0.17 to 0.55)* | 0.38 (0.18 to 0.56)* | 0.00 (-0.03 to 0.03) | 0.00 (-0.09 to 0.09) |
|  |  |  |  |  |  |  |
| Environmental context and resources | -3420.90 (-7970.12 to 1128.41) | 1.00 (1.00 to 1.00) | 0.39 (0.19 to 0.57)* | 0.38 (0.18 to 0.56)* | 0.00 (-0.02 to 0.06) | 0.01 (-0.10 to 0.17 ) |

All effects unstandardized with their 95% confidence intervals. The mediator-outcome effects are presented as odds ratios.

Professional role and identity as original percentage scores without transformation

*p= <0.05

ATE = average treatment effect; ADE = average direct effect; ACME = average causal mediation effect
